# Intrinsic Order and Disorder in the Bcl-2 Member Harakiri: Insights into Its Proapoptotic Activity

**DOI:** 10.1371/journal.pone.0021413

**Published:** 2011-06-23

**Authors:** Susana Barrera-Vilarmau, Patricia Obregón, Eva de Alba

**Affiliations:** Centro de Investigaciones Biológicas, Consejo Superior de Investigaciones Científicas, Madrid, Spain; University of South Florida College of Medicine, United States of America

## Abstract

Harakiri is a BH3-only member of the Bcl-2 family that localizes in membranes and induces cell death by binding to prosurvival Bcl-x_L_ and Bcl-2. The cytosolic domain of Harakiri is largely disorder with residual α-helical conformation according to previous structural studies. As these helical structures could play an important role in Harakiri's function, we have used NMR and circular dichroism to fully characterize them at the residue-atomic level. In addition, we report structural studies on a peptide fragment spanning Harakiri's C-terminal hydrophobic sequence, which potentially operates as a transmembrane domain. We initially checked by enzyme immunoassays and NMR that peptides encompassing different lengths of the cytosolic domain are functional as they bind Bcl-x_L_ and Bcl-2. The structural data in water indicate that the α-helical conformation is restricted to a 25-residue segment comprising the BH3 domain. However, structure calculation was precluded because of insufficient NMR restraints. To bypass this problem we used alcohol-water mixture to increase structure population and confirmed by NMR that the conformation in both milieus is equivalent. The resulting three-dimensional structure closely resembles that of peptides encompassing the BH3 domain of BH3-only members in complex with their prosurvival partners, suggesting that preformed structural elements in the disordered protein are central to binding. In contrast, the transmembrane domain forms in micelles a monomeric α-helix with a population close to 100%. Its three-dimensional structure here reported reveals features that explain its function as membrane anchor. Altogether these results are used to propose a tentative structural model of how Harakiri works.

## Introduction

**Figure 1:**
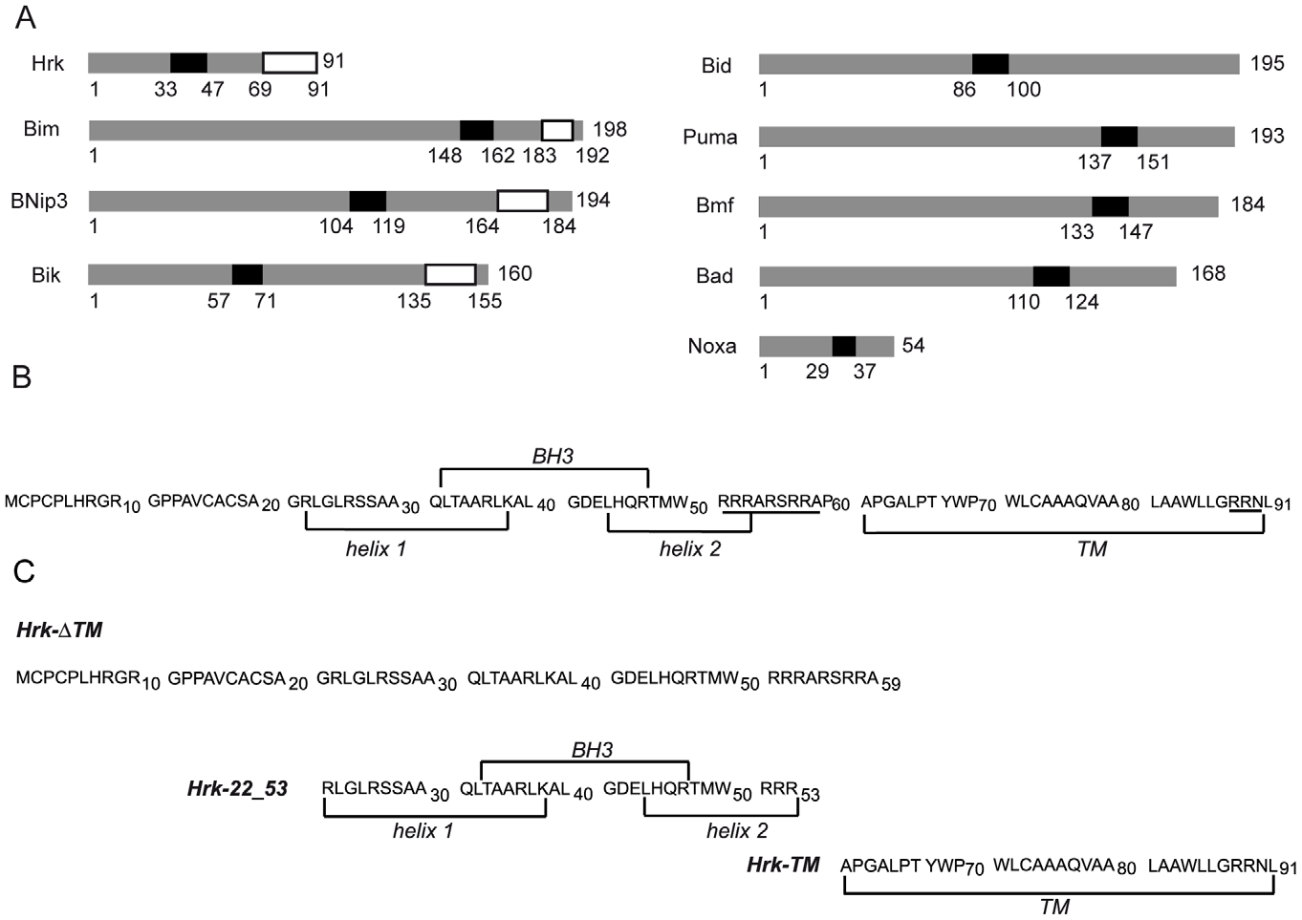
Members of the BH3-only subfamily and amino acid sequence of human Harakiri constructs. (A) Bars represent BH3-only proteins with size relative to the total amino acid sequence length. The position of the BH3 (black) and TM domains (white) is indicated. Members on the right do not show a TM domain. (B) Amino acid sequence of human Harakiri showing the position of the BH3 and TM domains. The predicted α-helices and the high number of Arg residues, characteristic of sequences preceding TM domains, are indicated. (C) Amino acid sequence of designed Harakiri constructs.

The form of programmed cell death, known as apoptosis, is essential for the correct development of multicellular organisms by inducing unwanted or damaged cells to commit suicide [1]. The dysfunction of apoptotic mechanisms is implicated in several pathologies including cancer, autoimmune and neurodegenerative disorders [2]. This cell death machinery is guarded by proteins of the Bcl-2 family, which promote and inhibit mitochondrial apoptosis [3], [4]. The prosurvival members (Bcl-2, Bcl-x_L_, Bcl-w, Mcl-1, A1) contain up to four Bcl-2 homology domains (BH1-BH4) together with a C-terminal hydrophobic sequence that typically directs them to the outer mitochondrial membrane. Prosurvival proteins can be antagonized by heterodimerizing with proapoptotic Bcl-2 members [5]–[8] that share homology solely in the BH3 region and are therefore grouped in the so-called BH3-only subfamily (**Fig. 1A**).

BH3-only proteins show significant structural diversity, including a single member (Bid) known to have a well-defined fold [9], [10], whereas others (i.e. Bim, Bad and Bmf) have been shown to be intrinsically unstructured [11]. In addition, several members are predicted to contain a TM domain (**Fig. 1A**). These characteristics turn structural studies on BH3-only proteins daunting and therefore scarce, despite their important role in cell death. For instance, only one study has been reported up to date addressing the role of the TM domain of a BH3-only protein (BNip3) at the atomic level. In this work, the three-dimensional (3D) structure of the TM domain of BNip3 was determined revealing the formation of a dimer of α-helices in the presence of lipid bicelles, thus suggesting a role in membrane permeabilization [12]. Kindred studies are necessary to help establish whether this is a common role in BH3-only TM domains.

Other structural studies have centered at complexes between prosurvival partners and peptides encompassing the BH3 region of BH3-only members. In all known 3D structures of these complexes the BH3 region forms an α-helix that is inserted in a hydrophobic groove of the antiapoptotic member [13]–[15]. These studies have recently raised significant interest because the reported structures serve as templates for the design of BH3 analogues to be used as drugs for cancer treatment [16]–[18]. However, little is known on the conformational preferences at atomic resolution of BH3-only proteins in isolation. Research in this direction can provide important insights into the mechanism of binding, thus strengthening the grounds for rational drug design. Moreover, such studies will help improve our understanding on the operating mode of functional intrinsically unstructured proteins, a field that is emerging as a new frontier in the concept of protein structure-function relationship.

Within the BH3-only subfamily we have focused our studies on the protein Harakiri (Hrk), which localizes in membranes in the cellular context and exerts proapoptotic activity by interacting with survival Bcl-x_L_ and Bcl-2 [19]. The BH3 region in Hrk, that forms part of its cytosolic domain, has been shown to be key for these interactions and thus for the killing activity [19]. In addition, Hrk possesses a stretch of approximately 30 residues at the C-terminus predicted as a TM domain [19]. Infrared studies on a peptide comprising this region have shown that it populates the α-helix conformation in the presence of lipids [20]. Most likely because of its toxicity, Hrk has proven to be difficult to produce by recombinant methods in other labs [11], [21] and ours. Thus, in our previous studies on the characterization of the interaction between Hrk and the Bcl-2 member Diva, the latter was produced recombinantly whereas we used for Hrk synthetic fragments encompassing the cytosolic domain [22]. These studies revealed that the cytosolic region can bind to Diva and that, at the structural level, is largely disordered with residual α-helical conformation at a population of ∼13% [22]. Hrk is therefore a potential model example combining both intrinsic disorder and membrane binding capabilities.

Up to date high-resolution structural studies on BH3-only proteins in isolation are lacking except for Bid [9], [10], the only known member with a defined fold. In an effort to improve our knowledge on the operating mode of BH3-only proteins we have characterized Hrk by NMR and circular dichroism (CD) to obtain atomic-detailed information on the conformational preferences of the cytosolic and TM domains. We have used synthetic constructs that encompass: 1) the entire cytosolic domain, 2) a smaller fragment including the BH3 motif and short flanking sequences at its N- and C-termini, 3) the TM domain. Firstly, we show using enzyme immunoassays and NMR that the cytosolic constructs are capable of binding Bcl-2 and Bcl-x_L_, the two proteins known to be implicated in Hrk's apoptotic function [19]. In addition, by combining previous structural data on the entire cytosolic domain [22] and new data on the smaller construct in water and water-alcohol mixture we find that α-helical propensity is confined in a 25-residue long fragment that comprises the 15 amino acid long BH3 motif. We have determined the 3D structure of the shorter cytosolic construct, revealing that it is strikingly similar to the structure of BH3-peptides bound to prosurvival proteins. This result suggests that intrinsic residual structure in disordered BH3-only proteins could play a fundamental role in the binding mechanism, as preformed conformations involving the BH3 domain are likely implicated in the interaction with survival partners.

Furthermore, we found that the TM domain of Hrk is poorly soluble in aqueous solution albeit it readily inserts into micelles. This result agrees with the reported localization of Hrk in membranes of intracellular organelles [19], suggesting that membrane binding is an important determinant of Hrk's function. We also show that the TM domain is monomeric in the presence of micelles, which points to a role as membrane anchor, in contrast to the TM domain of BNip3 found to function as a membrane permeabilization device [12]. Finally, we report the 3D structure of Hrk's TM domain in micelles revealing that it forms an α-helix followed by a well-ordered turn. The length of the helix and turn is ∼30Å, which is similar to the thickness of cellular membranes. The electrostatic surface of Hrk-TM structure is mainly apolar except for a small positively charged patch located at the C-terminal ordered turn, suggesting possible electrostatic interactions with the lipid heads of the membrane.

## Results

### Design of Hrk constructs and binding assays with Bcl-2 and Bcl-x_L_

Hrk was studied using synthetic fragments that together encompass the full-length protein (**Fig. 1B**). Its TM domain, as often times observed in other proteins, likely operates independently of the rest of the amino acid sequence, which could explain the reported localization in cellular membranes [19]. On these grounds we designed three synthetic constructs to perform structural and binding studies that could provide insight into the function of intact Hrk (**Fig. 1C**). Construct Hrk-ΔTM spans the entire cytosolic domain and construct Hrk-22_53 is a smaller fragment encompassing only the BH3 region and two flanking sequences predicted to form α-helices by the program PredictProtein [23]. Finally, construct Hrk-TM comprises the TM domain.

**Figure 2:**
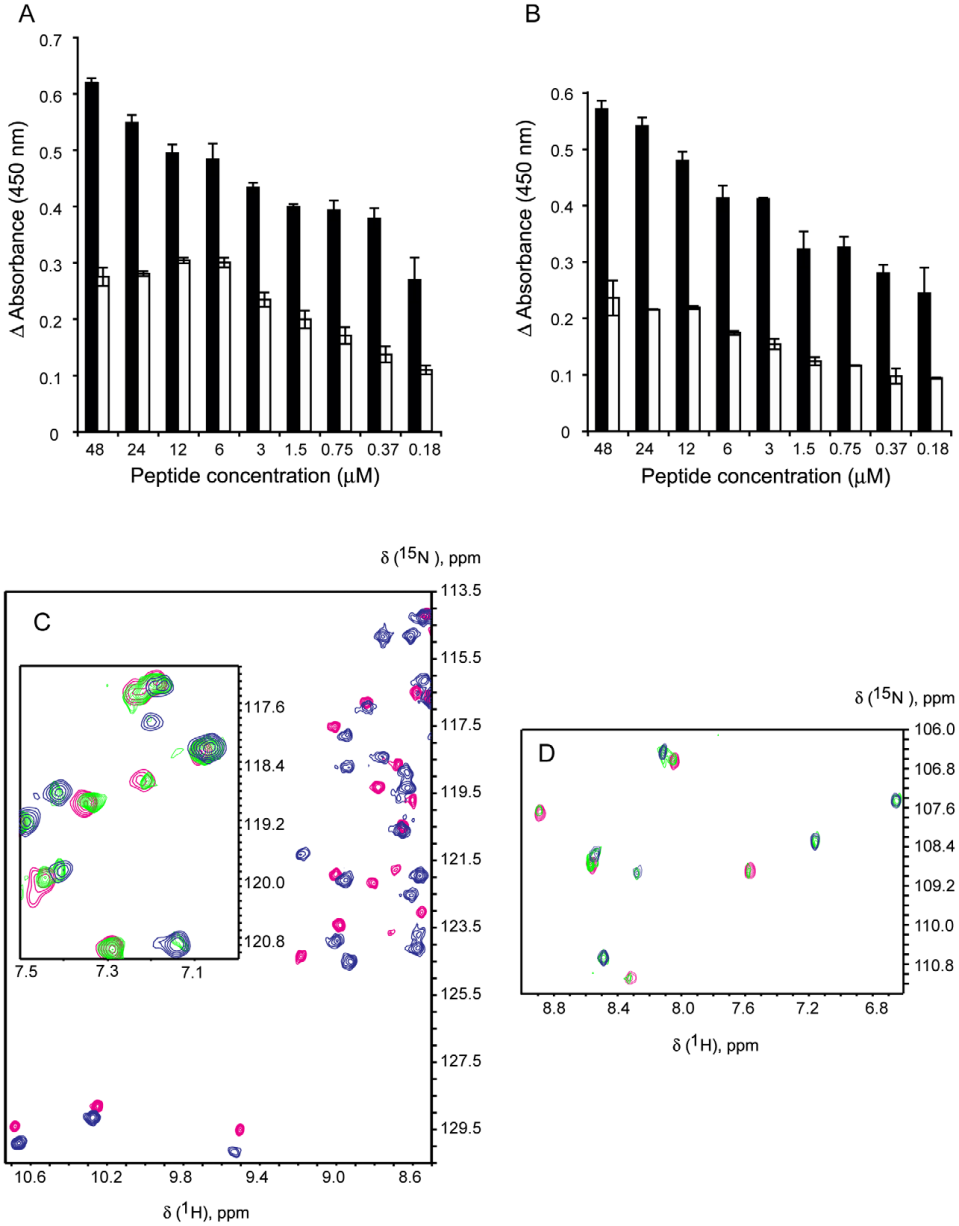
Binding of Hrk constructs to Bcl-2 and Bcl-x_L_ from ELISA and NMR. Difference in ELISA absorbance relative to the control for the binding of Bcl-2 (A) and Bcl-x_L_ (B) to Hrk-ΔTM (black bars) and Hrk-22_53 (white bars) vs. protein fragment concentration. Shown values are the average of two measurements. Thin bars represent the standard deviation. The absorbance value for the control is 0.098±0.002 and 0.122±0.008 for the experiment with Bcl-2 and Bcl-x_L_, respectively. Regions of [^1^H-^15^N]-HSQC spectra of free and complexed Bcl-x_L_ with Hrk-22_53 (C) and Hrk-ΔTM (D). The spectra in magenta correspond to unbound Bcl-x_L_. The blue and green spectra correspond to Bcl-x_L_/Hrk mixtures at ∼ 1:1 and 1:0.3 molar ratios, respectively. NMR signals of the green spectra overlap with signals of both the red and blue spectra, indicating slow conformational exchange in the chemical shift time scale.

Previous enzyme-linked immunosorbent assays (ELISA) and NMR binding data from our group report on the interaction between the cytosolic constructs of Hrk and the Bcl-2 member Diva [22]. These results indicate that the synthetic protein fragments are able to bind. However, the biological role of the Harakiri/Diva interaction is still to be studied. Thus, we analyzed the binding of Hrk-ΔTM and Hrk-22_53 to Bcl-2 and Bcl-x_L_ as these interactions have been reported to induce apoptosis [19]. Both cytosolic fragments show significant levels of interaction with the survival proteins relative to the control (**Fig. 2A**). These levels typically increase at higher peptide concentration as expected for real binding in ELISA. However, the binding levels of Hrk-22_53 for both Bcl-2 and Bcl-x_L_ do not increase beyond 12 µM (**Fig. 2A**). This result indicates that the two constructs show differences in binding. In fact, the entire cytosolic domain displays higher interacting levels than the shorter fragment, which suggests that the additional sequence of the longer construct can play a role in the interaction. Higher ELISA binding levels to Diva, confirmed by NMR, were also observed for the entire cytosolic domain relative to the shorter construct [22]. The ELISA data indicate that the cytosolic constructs of Hrk are able to bind prosurvival Bcl-2 members in the absence of the TM. Thus, both domains likely function independently.

The binding between Bcl-x_L_ and Hrk was also studied by NMR. The ^15^N-HSQC spectrum of unbound Bcl-x_L_ changes significantly by adding Hrk-22_53 at equimolar amount indicating direct interaction (**Fig. 2C**). An analogous result is obtained by the addition of Hrk-ΔTM (**Fig. 2D**). Moreover, the titration of Bcl-x_L_ with either Hrk-22_53 or Hrk-ΔTM leads to almost identical changes in the NMR spectra suggesting that the interaction is similar at the structural level. This behavior was also observed for the Diva/Hrk complexes mentioned above [22]. By increasing the concentration of the Hrk fragments in excess relative to Bcl-x_L_, no additional changes in the spectra were observed. This result indicates binding saturation at equimolar amounts of Bcl-x_L_ and the Hrk constructs, and the formation of 1∶1 Bcl-x_L_/Hrk-22_53 and Bcl-x_L_/Hrk-ΔTM complexes. Furthermore, two sets of NMR signals are present in the spectra of Bcl-x_L_/Hrk mixtures at 1∶0.3 molar ratio. One set corresponds to the spectrum of free Bcl-x_L_ and the second set to the complex, as inferred from signal overlap of the spectra at 1∶0.3 molar ratio with both the spectra of free Bcl-x_L_ and the one resulting from the equimolar mixture (**Fig. 2C, D**). This result indicates that exchange is in the slow regime relative to the chemical shift time scale. Binding saturation and slow exchange suggest high affinity constants for these complexes, in agreement with previous fluorescence data reporting a dissociation constant value of 92 nM for a complex between Bcl-x_L_ and a short 20 residue-long peptide comprising the BH3 domain of Hrk [24]. Nevertheless, further studies are necessary to understand the structural, dynamics and energetic factors implicated in the Bcl-x_L_/Hrk interaction.

### α-Helical structure in Hrk-ΔTM is localized in the region 29–53 encompassing the BH3 motif

Previous structural data from our group show that the cytosolic domain of Hrk is largely disordered [22]. In particular, small dispersion of amide ^1^H chemical shifts (**Fig. S1**) and NMR signals associated to methyl groups indicate that the protein is unfolded and lacks a hydrophobic core [22]. However, previous CD data on Hrk-ΔTM indicate the formation of approximately 13% population of α-helical structure. This population increases to 35% upon TFE addition at 35% (v/v) [22], a solvent known to promote secondary structure formation in protein fragments and peptides with intrinsic conformational propensity [25], [26].

To further characterize structurally Hrk-ΔTM by NMR is necessary to reduce signal overlap originating from both the small chemical shift dispersion resulting from the low structure population and the numerous signals of the rather long protein fragment. By augmenting the helical content is possible to increase chemical shift dispersion, as NMR signals are weight-averaged according to the population of the different conformations when they interconvert fast on the NMR experimental time. Unfortunately, NMR chemical shift dispersion in the presence of TFE is still small resulting in crowded spectra with severe signal overlap (**Fig. S1**), thus precluding further structural studies. Hrk-22_53 is a smaller fragment that will likely result in less complex spectra facilitating NMR analysis. However, it is important to check whether the helical propensity in Hrk-ΔTM is mainly located in the protein region flanking the BH3 motif (construct Hrk-22_53. **Fig. 1B, C**).

**Figure 3:**
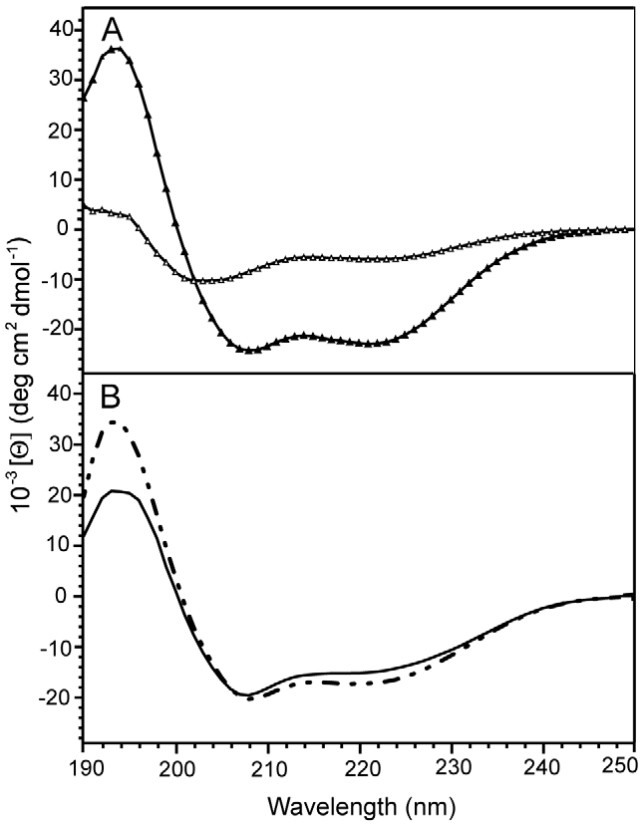
Far-ultraviolet circular dichroism (CD) spectra of Hrk constructs. (A) CD spectra of Hrk-22_53 in the presence (black triangles) and absence (white triangles) of TFE. (B) CD spectra of Hrk-TM in SDS/methanol (discontinuous line) and DPC/methanol (continuous line) mixed micelles.

The CD spectrum of Hrk-22_53 indicates a significant degree of disorder albeit with approximately 17% population of α-helical structure (**Fig. 3A**). This value is similar to that shown by Hrk-ΔTM [22], thus, taking into account the difference in peptide length, a simple calculation indicates that the helix spans a common region in both peptides. In other words, most helical population of Hrk-ΔTM is located in residues 22–53. A similar conclusion is derived by comparing the helical population of Hrk-22_53 and Hrk-ΔTM in the presence of TFE; 60% and 35% population respectively. However, the CD data indicate that both in aqueous milieu and in TFE, the helical content of Hrk-ΔTM is slightly larger than for the shorter fragment considering the difference in sequence length.

**Figure 4:**
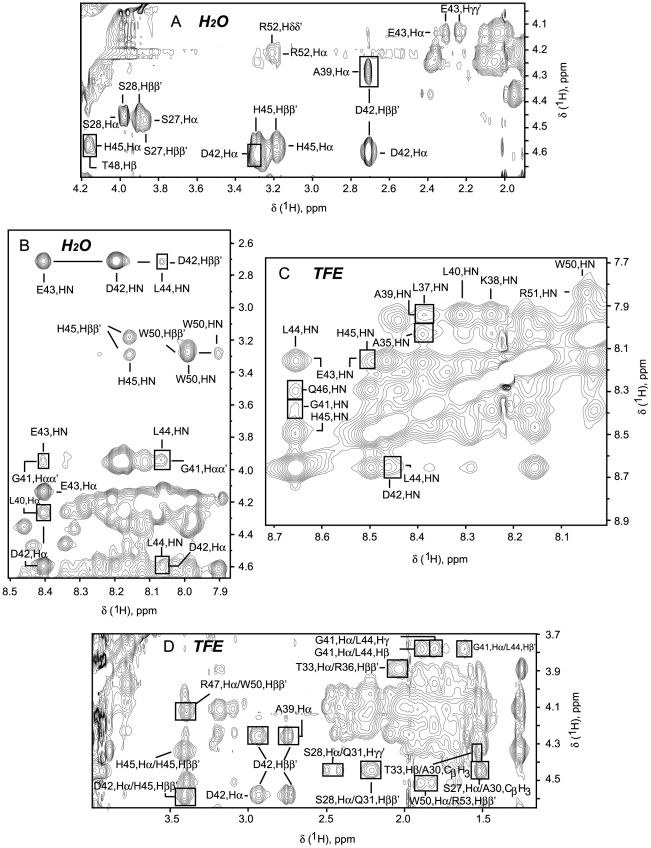
Selected regions of [^1^H-^1^H]-NOESY spectra of Hrk-22_53. (A, B) spectrum recorded in aqueous milieu. (C, D) spectrum recorded in 35% (v/v) TFE. NOE connectivities characteristic of the α-helical structure are boxed. For (C) H^N^-H^N^ (i, i+1) typical of the helix conformation are show but only H^N^-H^N^ (i, i+2) are boxed.

**Figure 5:**
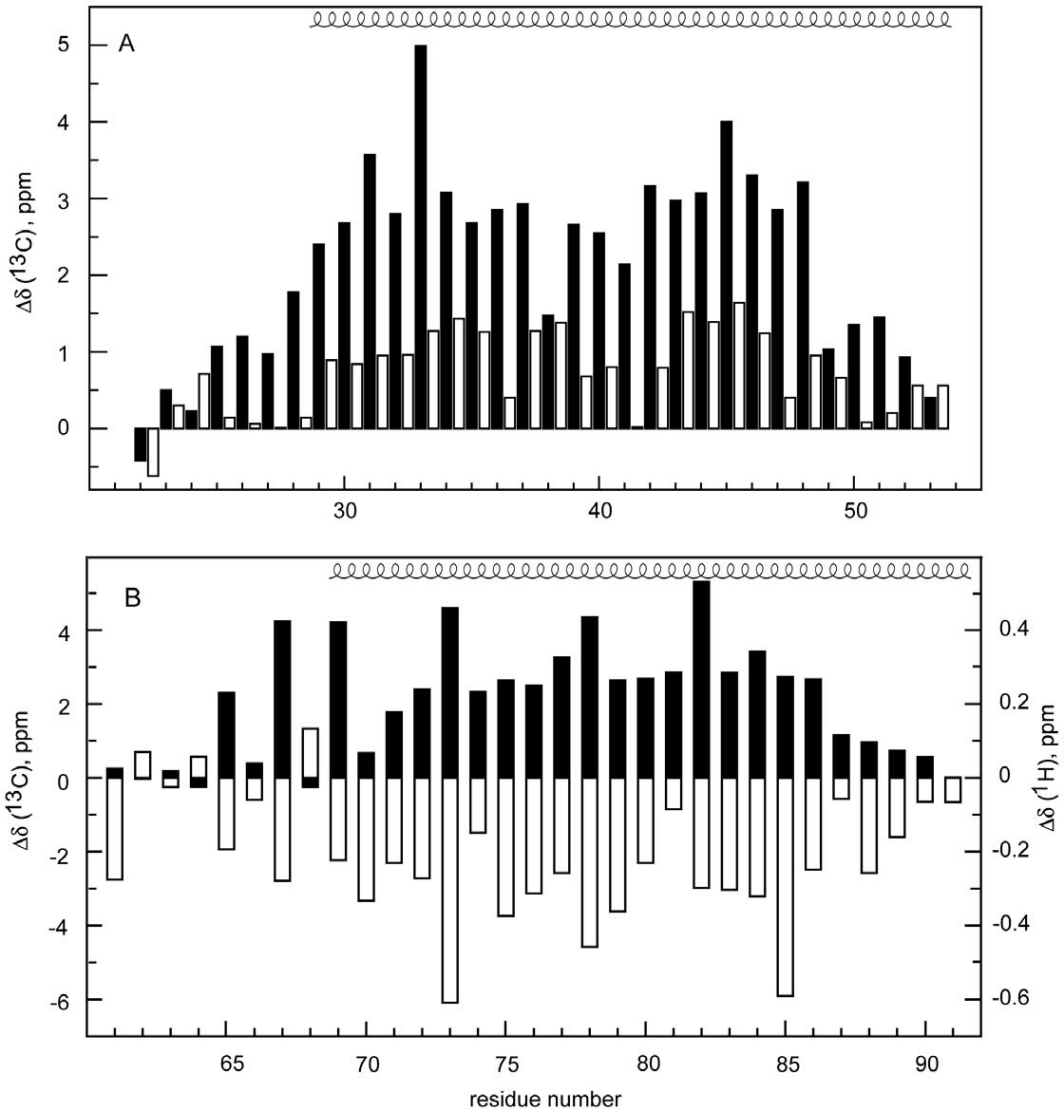
Secondary structure at the residue level of Hrk constructs from NMR data. (A) Difference between observed ^13^C_α_ chemical shifts and tabulated random coil values vs. residue number for Hrk-22_53 in the presence (black) and absence (white) of TFE. (B) Difference between observed ^13^C_α_ (black), ^1^H_α_ (white) chemical shifts and tabulated random coil values vs. residue number for Hrk-TM in SDS/methanol micelles. Helix-spanning residues are indicated on top of (A) and (B). ^1^H and ^13^C chemical shifts were obtained as explained in the Materials and Methods section. The random coil values used are those reported by Wishart et al. (1995) [27].

Hrk-22_53 results in less crowded NMR spectra (**Fig. S2A–D,** **Fig. 4**), thus allowing chemical shift assignment. As protein ^13^C_α_ and ^1^H_α_ chemical shifts are particularly sensitive to secondary structure, their deviation from values tabulated for unstructured peptides (random coil values) is routinely used to identify extended and helical conformations [27]. Chemical shifts differences relative to random coil values indicate that Hrk-22_53 adopts in water α-helical structure from residues 29 to 53 (**Fig. 5A** and **Fig. S3** for ^13^C_α_ and ^1^H_α_ deviations, respectively). This result contrasts with the prediction program [23], which suggests that the helix extends to residue 22 (**Fig. 1B**). The α-helix seems to bend around G41 as in this region the chemical shifts are closer to the random coil values (**Fig. 5A** and **Fig. S3**). Together with ^13^C_α_ and ^1^H_α_ chemical shifts, NOE data connecting local nuclei are also solid proof of the formation of protein secondary structure [28]. For α-helices the following NOEs are expected: H_α_-H_β_ (i, i+3), H_α_-H^N^ (i, i+1), H_α_-H^N^ (i, i+2), H_α_-H^N^ (i, i+3), H_α_-H^N^ (i, i+4), H^N^-H^N^ (i, i+1), H^N^-H^N^ (i, i+2) [28]. To obtain more detailed information on the structure formed by Hrk-22_53 in water NOE NMR experiments were recorded. For instance, H_α_-H_β_ (i, i+3) NOEs connecting Ala 39 to Asp 42, Asp 42 to His 45, and His 45 to Thr 48, indicate the formation of helical structure (**Fig. 4A**). Other NOE cross-peaks between protons H_α_-H^N^ (i, i+2) and (i, i+3) correlating residues Asp 42-Leu 44, Gly 41-Glu 43, Leu 40-Glu 43, Gly 41-Leu 44 (**Fig. 4B**) indicate α-helix formation centered at G41-D42, despite being the chemical shifts closer to random coil values than in other regions. In addition, H^N^-H^N^ (i, i+1) NOEs are observed from residues Thr 33 to Arg 52 (**Fig. S2E**). Altogether, the NOE data indicate that Hrk-22_53 adopts helical structure in water from residues Thr 33 to Arg 53 (**Fig. 4A,B** **, Fig. S2E**), in very good agreement with the information obtained from chemical shifts (**Fig. 5A** and **Fig. S3**).

The chemical shift and NOE data unambiguously show that the cytosolic domain of Hrk forms an α-helix in water in the region 29–53. However, because of the low structure population and because some NOEs cannot be observed due to chemical shift overlap, the number of NOE connectivities is insufficient to reach an ensemble of conformers representing the helix when included in the structure calculation protocol (data not shown), resulting in ∼3Å r.m.s.d for the 20-conformer ensemble. In contrast, by TFE addition the ^1^H_α_ and ^13^C_α_ chemical shifts of Hrk-22_53 show larger deviations from random coil values as expected for a larger structure population (**Fig. 5A** **, Fig. S3**). The profiles of ^1^H_α_ and ^13^C_α_ chemical shift deviations with and without TFE are equivalent, which indicates that TFE increases the population in the region with intrinsic propensity without inducing significant helix formation in other segments. In addition, NOE peaks observed in water and in TFE that are characteristic of the α-helix are analogous (**Fig. 4**). However, these peaks are more intense and abundant in water/alcohol mixture as a result of the larger population (**Fig. 4C, D**). The chemical shift and NOE comparison in water and TFE indicates that the structure is equivalent in both media albeit with different population. The larger population in TFE led to more NMR restraints resulting in a single ensemble of conformers. The structural details are explained below.

### The 3D structure of unbound Hrk-22_53 in TFE closely resembles BH3-peptide structures complexed to prosurvival Bcl-2 members

**Figure 6:**
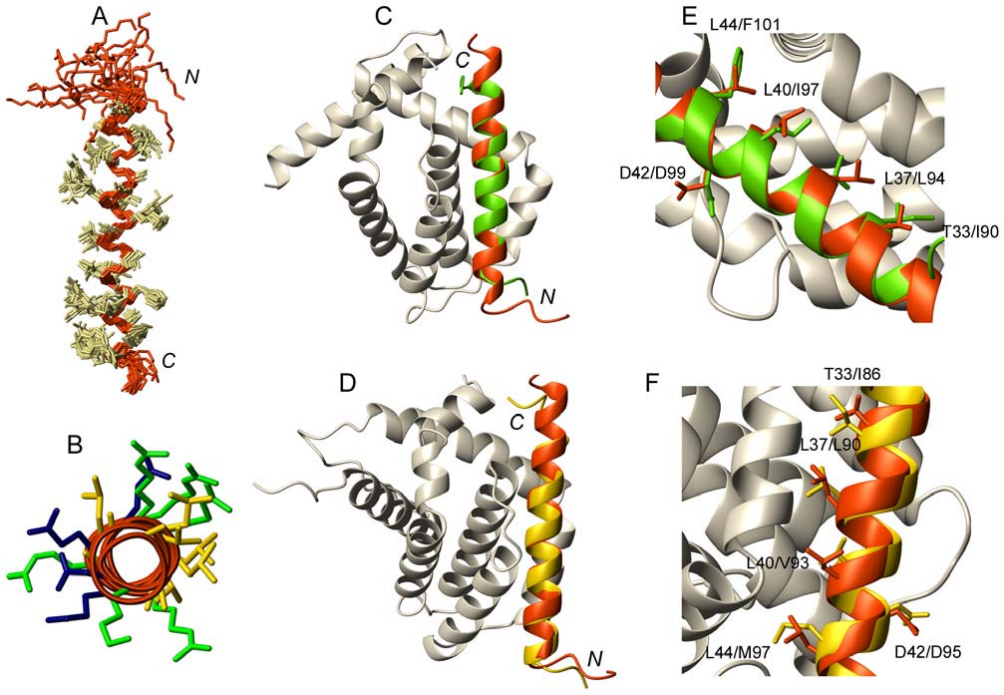
Three-dimensional structure of the cytosolic fragment of Hrk in TFE (residues 22–53) compared to BH3-peptides in heterodimer structures. (A) Backbone superposition of the 20 conformers with lowest energy. Side chains are shown in ivory. N and C termini are indicated. (B) Top view of the helix formed by Hrk-22_53 showing in yellow the clustering of hydrophobic residues Leu and Ala flanked by polar residues Lys, Arg (in green) and Gln, Glu and Asp (in blue). (C, D) Superposition of Hrk-22_53 (red) and the helix formed by a peptide comprising the BH3 domain of the BH3-only protein Bid (green) in complex with prosurvival Mcl-1 (gray, PDB ID 2KBW) (C) and the BH3 domain of the BH3-only protein Bim (yellow) in complex with prosurvival Bcl-x_L_ (gray, PDB ID 3FDL) (D). (E, F) Side chain position of the four conserved hydrophobic residues anchoring the helix to the prosurvival protein groove and highly conserved Asp in Hrk-22_53 (red), Bid-BH3-peptide (green, PDB ID 2KBW) (E), Bim-BH3 peptide (yellow, PDB ID 3FDL) (F). Residues are labeled.

Structural information at atomic resolution on the unbound form of BH3-peptides or intrinsically disordered BH3-only proteins has not been reported to date. However, this information is important to better understand the determinants of BH3-only protein binding. The comparison of the structure of Hrk-22_53 to the known structures of BH3-peptides complexed with their prosurvival partners could provide insights into this subject. To this aim we have calculated the solution structure of Hrk-22_53. Structural statistics of the resulting ensemble of the 20 conformers with lowest energy are shown in **Table S1**. This ensemble forms an α-helix spanning residues 29–51 with significantly well defined backbone (r.m.s.d = 0.5Å) and side chains (**Fig. 6A**). In contrast, the absence of a single conformation for residues 22–28 indicates that this region is disordered and residues 52–53 also appear to be more flexible (**Fig. 6A**). One side along the longitudinal axis of the α-helix is mainly hydrophobic, albeit surrounded by a significant number of positively (Arg, Lys) and negatively charged residues (Asp, Glu) forming an amphipathic helix (**Fig. 6B**). The apolar face of the helix most likely will interact with the hydrophobic groove of the antiapoptotic partner upon complex formation, leaving the polar side exposed to the solvent.

The structure of unbound Hrk-22_53 is compared to two reported BH3-peptide/prosurvival complex structures (Mcl-1/Bid-BH3 [15] and Bcl-x_L_/Bim-BH3 [29]) in **Fig. 6C–F**. Although the length of the different helices formed by the BH3-peptides varies, the structure of Hrk-22_53 can be superimposed to these helices using as reference two highly conserved residues in the BH3 domain of the three proteins (Gly 41 and Asp 42 in Hrk) (**Fig. 6C, D**). According to the reported 3D structures of the survival/BH3-peptide complexes, 4 conserved hydrophobic residues in the BH3 domain of the BH3-peptides form part of the interface and help to anchor the peptide to the survival protein hydrophobic groove [13]. The superposition of the structures in **Fig. 6E, F** shows that the side chain position of these conserved residues (Thr 33, Leu 37, Leu 40 and Leu 44 in Hrk) forming part of the interface is equivalent in Hrk-22_53 and the bound forms of Bid-BH3 and Bim-BH3.

The structures of unbound Hrk and the complexed peptides are significantly similar. This result suggests that the preformed helical conformation can play an important role in the binding process. In fact, previous studies on peptides derived from the BH3 domain of the BH3-only protein BAD have shown that affinity towards Bcl-x_L_ increases by augmenting the α-helical content through mutations [14]. It is currently unknown whether the α-helical population in isolation might increase by folding upon binding once the partner is present, or only the preformed helices are able to bind shifting in turn the folding equilibrium towards the structured form. At this point it is worth to recall the higher binding levels of the long Hrk construct to Diva [22], Bcl-2 and Bcl-x_L_. However, the NMR and ELISA studies on the Diva/Harakiri interaction suggest that the interacting surface in Diva is structurally similar when complexed to both Hrk-ΔTM and Hrk-22_53 [22]. These results indicate that the additional sequence of the longer construct is influencing binding albeit without modifying the interacting region. The unstructured sequences flanking the helix could enhance binding by forming transient contacts with the prosurvival protein or by augmenting the helical propensity of the binding region in Hrk. In fact, the entire cytosolic domain shows larger α-helical population according to the CD data (vide supra).

The factors involved in the binding of intrinsically disordered proteins to their partners are still poorly understood. Recent findings indicate that this mechanism is highly complex and can vary greatly depending on the intrinsically disordered protein studied [30]. For instance, several or all of the following; preformed elements of secondary structure, tertiary contacts between the interacting partners and the dynamics of the bound and unbound forms, can be key players in the binding process [30]–[33]. In this context the behavior of Hrk is different from other intrinsically disordered proteins that do not adopt preformed structural elements [34], or when these are present they do not match the final structure adopted upon complex formation [30].

### 3D structure of Hrk-TM in micelles

In an effort to characterize the solution structure of Hrk-TM at atomic resolution we have tested different conditions that could render both high-quality NMR spectra and emulate the membrane environment. Hrk-TM is barely soluble in aqueous solution as expected from the hydrophobic nature of its amino acid sequence (**Fig. 1C**), thus precluding NMR studies in water. However, it readily dissolves in the presence of micelles formed by SDS and DPC detergents. In both micellar systems NMR signals are significantly broad complicating NMR analysis. However, spectra quality is slightly better in SDS. It has been reported that the addition of alcohol to detergent micelles increases micelle flexibility, as alcohol molecules apparently interact with the detergent hydrophobic chains affecting their mobility [35], [36]. Higher micelle flexibility can result in narrower NMR signal linewidth and therefore Hrk-TM was dissolved in SDS micelles mixed with the simplest alcohol, methanol. Under these conditions the resulting NMR spectra were suitable for analysis. However, SDS and DPC are detergents with significantly different chemical properties, thus CD experiments were performed first to test whether the conformational behavior of Hrk-TM varies depending on the micellar system used. The CD data indicate that Hrk-TM adopts similar structure in both milieus, corresponding to ∼60% population of α-helix in SDS/methanol and ∼55% in DPC/methanol micelles (**Fig. 3B**).

**Figure 7:**
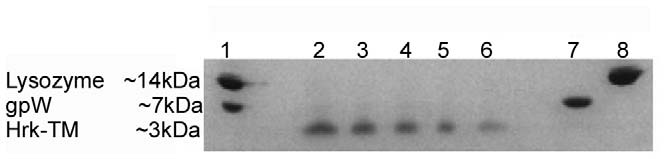
Oligomerization state of Hrk-TM in micelles. SDS-polyacrylamide gel of mixed Lysozyme (∼14 kDa) and gpW (∼7 kDa) in well 1 and in different solutions in wells 7 and 8, together with Hrk-TM (∼3 kDa) at decreasing concentrations: 0.5 mM, 0.33 mM, 0.25 mM, 0.2 mM, 0.16 mM in wells 2**–**6.

Once checked that Hrk-TM is able to form a large population of α-helical structure in micelles, the next step was to investigate the oligomerization state in this medium. It is well known that TM α-helices migrate in SDS-polyacrilamide gel electrophoresis (SDS-PAGE) according to their native-like oligomeric sate [37], [38]. Therefore, the migration of Hrk-TM in SDS-PAGE was compared to other proteins of known molecular weight (**Fig. 7**). Our results indicate that Hrk-TM migrates in SDS-PAGE according to its molecular weight (∼3 kDa), thus faster than the two proteins (Lysozyme and gpW) used in the experiment with molecular weight of ∼14 kDa and ∼7 kDa, respectively. Thus, Hrk-TM is monomeric in the presence of SDS micelles.

NMR can be used to get information on whether TM domains get inserted in the micellar environment. For instance, proteins in aqueous solution typically show in [^1^H-^1^H]-NOESY spectra cross-peaks at amide proton and water chemical shifts, which originate from chemical exchange between them (**Fig. S4**). The absence of these cross-peaks indicates that amide protons are protected from the solvent, for example by the surrounding detergent molecules [37]. Only Asn 90 at the C-terminus of Hrk-TM shows a NOE cross-peak between its amide proton and water in the NOESY spectra of Hrk-TM in micelles (**Fig. S4**). This result indicates that Hrk's TM domain penetrates the micelle.

The ^1^H_α_ and ^13^C_α_ chemical shifts of Hrk-TM in the micellar environment consistently indicate the formation of helical structure spanning residues Trp 69 to Leu 91 (**Fig. 5B**). However, no regular secondary structure is observed from residues Ala 61 to Tyr 68. The population of α-helix in solution can be estimated from the magnitude of C_α_ chemical shifts relative to tabulated random coil values [39]. Excluding the 8 N-terminal residues, which do not adopt regular secondary structure, the α-helical population estimated from NMR data is 95%. However, when all 31 residues are included the population drops to 61%, which is in very good agreement with the population calculated from the CD data that, in contrast to NMR, can only report information averaged for all residues.

**Figure 8:**
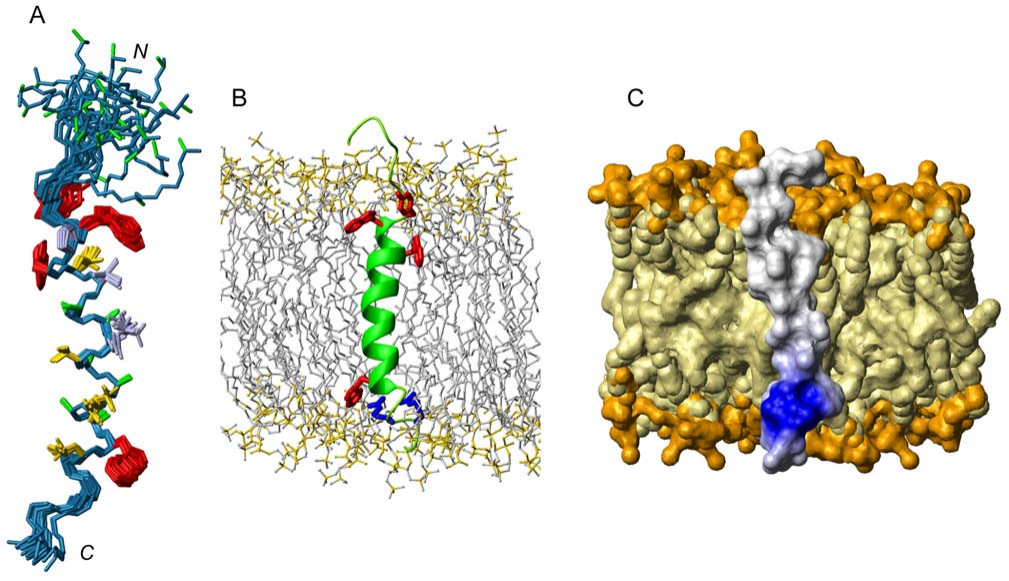
Three-dimensional structure of the TM domain of Hrk. (A) Superposition of the 20 conformers of Hrk-TM with lowest energy. Trp and Tyr residues are colored in red, Leu and Val in yellow, Ala in green, other residues in gray. (B) Ribbon representation of the structure of Hrk-TM placed in a lipid bilayer resulting from molecular dynamics simulation of POPC (Palmitoyl oleoyl phosphatidyl choline) [57]. Aromatic residues (Trp and Tyr) are colored in red and Arg residues in blue. The apolar hydrocarbon chains and polar heads of the lipids are colored in gray and yellow, respectively. (C) Electrostatic surface representation of Hrk-TM placed in the lipid bilayer. Apolar and positively charged surfaces in Hrk-TM are colored in gray and blue. The apolar surface and the charged heads of the bilayer are colored in light and dark yellow.

The NMR structure of Hrk-TM (structural statistics in **Table S2**) shows that the α-helix actually spans residues Trp 69 to Leu 85 and is followed by a well-ordered turn from residues Leu 86 to Leu 91 (**Fig. 8A**). The backbone and in particular the side chains of aromatic residues are very well defined (**Fig. 8A** and **Table S2**). The helix is slightly curved showing in its concave part bulky hydrophobic residues such as Leu and Val, and in the convex part less hydrophobic or bulky amino acids (Ala, Gln or Cys) (**Fig. 8A**). The length of the helix including the well-ordered turn is 31Å, which is very close to the thickness of the hydrophobic inner part of a lipid bilayer. This result suggests that Hrk-TM could fully span the bilayer leaving outside the N-terminal sequence that does not form part of the helix. Aromatic residues, specifically Trp, are particularly abundant in protein TM domains and are not evenly distributed throughout the domain, rather are concentrated at the edges [40]. The role of these hydrophobic and bulky residues is believed to prevent the TM helix from slipping across the membrane. Hrk-TM contains 3 Trp and 1 Tyr. To illustrate the position of these residues in the membrane context the structure of Hrk-TM is placed on a model of a lipid bilayer on **Fig. 8B**. As these studies were done in micelles, the precise angle that the long axis of the TM helix forms with the normal of the lipid bilayer is not known. However, NMR data on amide ^1^H protection from the solvent suggest that the TM domain would be fully inserted in the membrane. Additionally, the two Arg residues at the C-terminus (**Fig. 8B**) are unlikely buried in the hydrophobic part of the bilayer, as this would require a high energetic cost. These residues form a positively charged patch in the otherwise apolar electrostatic surface of Hrk-TM that could potentially form electrostatic interactions with the polar heads of the bilayer. These results suggest that Hrk-TM likely adopts a position close to parallel to the bilayer normal as shown in **Fig. 8B, C**. Summarizing, the monomeric state of Hrk-TM and its structural characteristics suggest that it functions as a membrane-anchoring device.

## Discussion

This study reveals high-resolution structural characteristics of both the cytosolic and the TM domain of the Bcl-2 protein Hrk. The cytosolic domain is largely unstructured in solution [22], which indicates that Hrk shares features commonly found in intrinsically disordered proteins. Three other members of the BH3-only subfamily have been shown to be unstructured [11]. Thus, intrinsic disorder most likely plays an important role in the function of these proteins. However, no structural characterization at atomic resolution of a natively unfolded Bcl-2 member has been reported up to date. In the case of Hrk, our study shows that out of the 59 residues in the cytosolic domain only a stretch of 25 (residues 29–53), including the BH3 region, significantly populates the α-helical conformation. This helical structure very closely resembles the α-helix formed by BH3-peptides in complex with survival partners, which suggests that preformed elements of secondary structure are major players in the binding mechanism. This result agrees with recent structural studies on Protein Phophatase 1 regulators indicating that the transient α-helix conformation likely plays a key role in binding [41]. However, other intrinsically disordered proteins that fold upon binding do not adopt residual secondary structures in isolation [34].

In addition, the low solubility of Hrk-TM in aqueous milieu and its capability of forming ∼100% populated helical structure in micelles agree with previous reports indicating the localization of Hrk in intracellular organelle membranes [19]. It is worth noting that Hrk is predicted to have an N-myristoylation site at Gly 63 (at the N-terminus of Hrk-TM) according to prediction programs [23]. Myristoylation is known to play a vital role in membrane targeting. Furthermore, the monomeric state of Hrk-TM in micelles and its 3D structure suggest that it likely functions as a membrane anchor. Up to date high-resolution structural information on the TM domain of BH3-only proteins besides Hrk-TM is known only for BNip3, which has been suggested to function as a membrane permeabilization device [12]. The disturbance of the mitochondrial membrane is a recognized mechanism to trigger cell death, therefore the different behavior of Hrk and BNip3, together with the structural heterogeneity found in the BH3-only subfamily point to a wide variety of mechanisms to prompt apoptosis in this subfamily.

**Figure 9:**
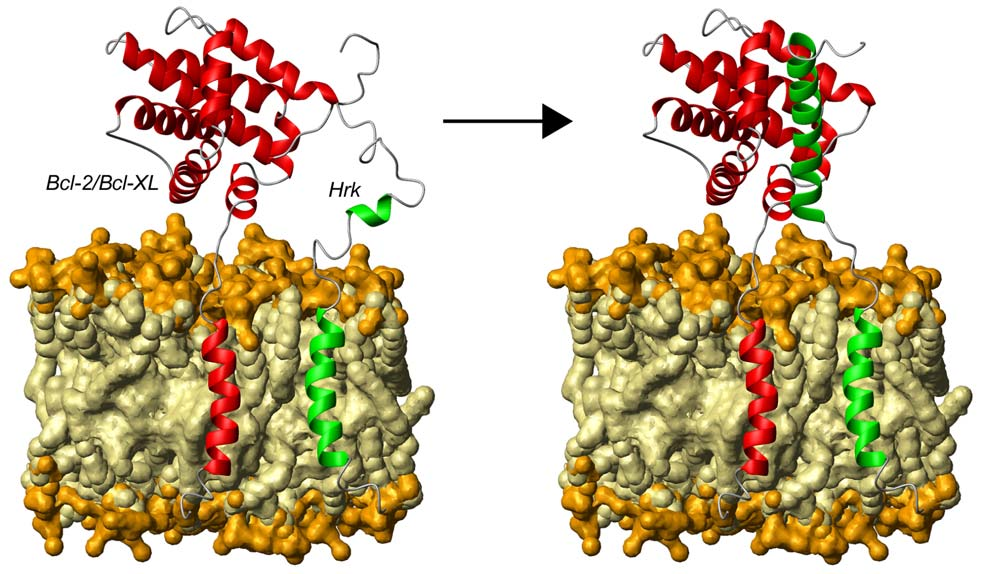
Tentative structural model of Harakiri's operating mode. The survival protein (Bcl-2 or Bcl-x_L_) [13], [14] and Hrk are represented as red and green ribbons, respectively. The arrow connects the states before (Hrk's cytosolic domain is shown largely disordered with preformed secondary structure) and after (Hrk's cytosolic domain is forming a helix) the interaction between Hrk and the survival partner. The hydrophobic and polar parts of the bilayer are colored in light and dark yellow, respectively.

Finally, ELISA and NMR data indicate that the cytosolic domain of Hrk can bind Bcl-2 and Bcl-x_L_ independently of the TM domain, which according to our structural results functions as an anchoring device. Based on these considerations and combining the results on both domains, we propose a tentative structural model to help explain how Hrk works. According to this model, Hrk is anchored to the mitochondrial membrane through its TM helix exposing the disordered N-terminus to the cytosol (**Fig. 9**). This domain folds into an α-helix from residues 29–53 upon binding to the prosurvival partner that could be already anchored to the membrane or in the cytosol. The intrinsic flexibility of the disordered domain likely facilitates binding by increasing the capture radius for prosurvival Bcl-2 members. The advantageous effect of flexibility in the binding of intrinsically disordered proteins has been theoretically predicted before as a “fly-casting” mechanism [31], and suggested for multi-domain proteins with long semi-flexible linkers and protein-binding function based on structural and dynamics studies [42]. This tentative mode of operation for Harakiri agrees with the recently proposed “membrane-embedded-together” model for apoptotic mechanisms [43], which emphasizes that many interactions within the Bcl-2 family occur only in the membrane.

## Materials and Methods

### Peptide synthesis

The cytosolic domain of human Hrk, residues 1–59 (Hrk-ΔTM), the construct comprising residues 22–53 (Hrk-22_53), and the fragment spanning the TM domain, residues 61–91 (Hrk-TM) (**Fig. 1C**) were synthesized and purified by CASLO Laboratory (Denmark). The purity (>95%) and molecular weight were confirmed by liquid chromatography and mass spectrometry, respectively. Hrk-22_53 and Hrk-TM are protected by a C-terminal amide.

### Bcl-x_L_ cloning, expression and purification

A human Bcl-x_L_ construct comprising residues 1–209 (including the flexible ∼50 residue-long loop) was cloned by TopGen Technologies (Canada) into a pET21a vector (Novagen) between the NdeI and NotI restrictions sites, thus without the histidine tag. The protein was expressed in C41(DE3) E. Coli strain during 3 hours at 37°C by the addition of isopropyl β-D-thiogalactopyranoside (1 mM concentration) at an OD_600_ of 0.6–0.7. Uniformly ^15^N-labeled Bcl-x_L_ was produced using ^15^NH_4_Cl (Cambridge Isotopes) as sole nitrogen source. The cells were harvested by centrifugation and resuspended in a buffer containing 10 mM Tris_HCl at pH 7.0, 0.1 mM protease inhibitor cocktail (Sigma) and 1 mM TCEP (Tris(2-carboxyethyl)phosphine). Cells were lysed by sonication at 4°C and centrifuged at 25,000 rpm for 1 hour. The soluble protein was purified by anion exchange chromatography using a HiTrap Q sepharose Fast Flow column (GE Healthcare), followed by reverse phase chromatography (C8 column) in water-acetonitrile mixtures and lyophilization of the protein solution. Mass spectrometry was used to confirm the ^15^N-labeled Bcl-x_L_ sample (residues 1–209).

### Enzyme-linked immunosorbent assays (ELISA)

Microplates (Costar Ltd., US) were coated with 50 µl of Bcl-2 or Bcl-x_L_ both lacking the TM domain (purchased to R&D Systems, Inc., US) at a concentration of 7.5 µg/ml in PBS and incubated overnight at 4°C. Plates were then washed three times with distilled water and blocked with 2.5% BSA (Sigma) in PBS for 2 hours at 37°C. Stock solutions of Hrk constructs (Hrk-ΔTM and Hrk-22_53) were prepared at fixed initial peptide concentrations derived from absorbance measurements at 280 nm. The final peptide solutions used in the binding studies contained PBS, 2.5% BSA and 0.1% Tween to avoid aggregation and non-specific binding. Hrk samples were loaded and incubated overnight at 4°C. BSA at 2.5% in PBS was used as negative control. After thorough wash with distilled water and 0.1% Tween, a polyclonal rabbit anti-Harakiri BH3 domain antiserum was added (1∶1000) (Abcam, UK). Following a streptavidin peroxidase conjugate anti-rabbit Ig (1∶1000) (Dako) as secondary antibody, the peroxidase activity was detected by the addition of 3,3′,5,5′-tetramethylbenzidine dihydrochloride peroxidase substrate (Sigma). Colour development was then allowed to proceed for 15 minutes at room temperature and stopped adding 0.1 M H_2_SO_4_. The optical density was read at 450 nm on an ELISA plate reader (Labsystems Multiskan BICHROMATIC). All assays were done in duplicate. The absorbance of the control at 450 nm (∼0.1) is within typical background values in ELISA experiments.

### NMR spectroscopy

Hrk-ΔTM and Hrk-22_53 NMR samples were prepared at ∼1.0–1.5 mM peptide, 20 mM phosphate buffer, 5 mM d_16_-TCEP (only for Hrk-ΔTM), 0.1 mM NaN_3_, pH 5.8, 5% D_2_O/H_2_O or 100% D_2_O, in the absence and presence of 35% (v/v) d_2_-TFE or d_3_-TFE for fully deuterated solvent. Hrk-TM was dissolved at ∼1.0 mM peptide concentration in 200 mM d_25_-SDS or 200 mM d_38_-DPC, 5 mM d_16_-TCEP, 0.1 mM NaN_3_, pH 3.2, 5% D_2_O/H_2_O or 100% D_2_O, in the presence of 35% (v/v) d_3_-methanol or d_4_-methanol for fully deuterated solvent. NMR experiments were acquired at 303 K for Hrk-ΔTM and Hrk-22_53, and 323 K for Hrk-TM in a Bruker Avance III 600 MHz spectrometer equipped with a triple-resonance three-axis gradient probe and an Avance 600 MHz magnet equipped with a cryoprobe. NMR chemical shift assignments were obtained from the experiments: TOCSY at mixing times of 80 ms for Hrk-ΔTM and Hrk-22_53, and 70 ms for Hrk-TM; NOESY at mixing times of 250 ms for Hrk-ΔTM and Hrk-22_53, and 150 ms for Hrk-TM. Chemical shifts of ^13^C nuclei for Hrk-TM were obtained from [^1^H-^13^C]-HSQC [44], [45] at ^13^C natural abundance following the ^1^H chemical shift assignment resulting from the TOCSY and NOESY experiments. However, ^13^C chemical shifts of Hrk-22_53 in the absence and presence of TFE were obtained by combining data from [^1^H-^13^C]-HSQC and 3D [^1^H-^1^H]-TOCSY-[^1^H-^13^C]-HSQC [46] at ^13^C natural abundance in a cold probe to alleviate signal overlap. Experiments were processed with NMRPipe [47] and analyzed with PIPP [48] and Sparky [49].

### Titration of Bcl-x_L_ with Hrk-22_53 and Hrk-ΔTM by NMR

Stock solutions of ^15^N-labeled Bcl-x_L_ were prepared at protein concentrations of 0.3 and 0.24 mM in the presence of 0.1 mM NaN_3_, 5 mM d_16_-TCEP, pH 10.0. Hrk-22_53 and Hrk-ΔTM stock solutions were prepared at 2 mM and 1.7 mM, respectively. Small aliquots of Hrk-22_53 and Hrk-ΔTM stock solutions were added to the Bcl-x_L_ samples to reach the following approximate final concentrations: Bcl-x_L_/Hrk-22_53, 0.3 mM/0.0 mM; 0.28 mM/0.09 mM (1∶0.3 molar ratio); 0.27 mM/0.21 mM (1∶0.8 molar ratio); 0.23 mM/0.46 mM (1∶2 molar ratio); Bcl-x_L_/Hrk-ΔTM, 0.24 mM/0.0 mM; 0.23 mM/0.08 mM (1∶0.3 molar ratio); 0.20 mM/0.21 mM (1∶1 molar ratio). [^1^H-^15^N]-HSQC spectra were acquired for each Bcl-x_L_/Hrk mixture at 303 K to follow chemical shift changes of ^15^N-labeled Bcl-x_L_ upon the interaction with the unlabeled Hrk constructs.

### Structure calculation

Peak intensities from NOESY experiments on Hrk-22_53 with and without TFE were translated into a continuous distribution of interproton distances. A summation averaging ((∑r^−6^)^−1/6^) was used to represent distances involving methyl groups, aromatic ring protons and non-stereospecifically assigned methylene protons [50]. Errors of 35% of the distances were applied to obtain lower and upper limits. Distance restraints for a total of 17 hydrogen bonds (r_NH-O_ = 1.9–2.5Å, r_N-O_ = 2.8–3.4Å) were defined according to the experimentally determined secondary structure of the protein only for Hrk-22_53 in TFE. The TALOS program [51] was used to obtain 38 and 18 dihedral restraints for Hrk-22_53 with and without TFE, respectively, for those residues with statistically significant predictions. Peak-intensities from NOESY experiment on Hrk-TM in micelles were classified in 4 categories: strong (2.2Å, −0.3, +0.7), medium (3.0Å, −0.9, +0.9), weak (3.5Å, −1.1, +1.1) and very weak (4Å, −1.0, +1.0). Distance restrains corresponding to 12 hydrogen bonds derived from the secondary structure were used. φ and ψ dihedral angle restraints for 16 residues were derived from statistically significant TALOS [51] predictions. Structures were calculated with the program X-PLOR-NIH 2.16.0 [52]. The starting structure was heated to 3,000 K and cooled in 20,000 steps of 0.002 ps during simulated annealing. The final ensemble of 20 NMR structures was selected based on lowest energy and no restraint-violation criteria. The 20 lowest-energy conformers have no distance restraint violations and no dihedral angle violations greater than 0.3Å and 3°, respectively for Hrk-22_53 in TFE, 0.4Å and 5.5°, respectively for Hrk-22_53 without TFE, 0.5Å and 5°, respectively, for and Hrk-TM in micelles. Structures were analyzed with MOLMOL [53] and their quality was assessed with PROCHECK-NMR [54]. Coordinates for Hrk-22_53 in TFE and Hrk-TM in SDS/methanol micelles were deposited in the Protein Data Bank with accession codes 2l58 and 2l5b, respectively.

### Circular dichroism

Hrk-22_53 was dissolved in 20 mM sodium phosphate buffer at pH 5.8 in the presence and absence of 35% (v/v) trifluoroethanol (TFE). The concentration of Hrk-22_53 was 36 µM in the buffer without TFE and 37 µM in TFE, respectively. Hrk-TM was dissolved in 200 mM SDS, 35% (v/v) methanol, pH 3.2 and 200 mM DPC, 35% (v/v) methanol, pH 3.2 at 50 µM and 54 µM concentration, respectively. Concentration values were calculated by measuring absorbance at 280 nm. Circular dichroism measurements were acquired at room temperature using a JASCO model J-815 spectropolarimeter with a 1 mm cuvette. The CD signal at 222 nm was converted to mean residue ellipticity ([θ]*_obs_*) after subtracting the blank using the equation:
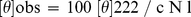
(1)where *c* is the peptide concentration (in mM), N is the number of peptide residues and *l* is the path-length (in cm).

The percentage of α-helical population was determined using the following equation:
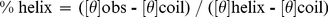
(2)where [θ]*_helix_* is the mean residue ellipticity of a complete helix, i.e., -42,500(1-(3/N)), and [θ]*_coil_* is the ellipticity of a random coil, i.e. +640 [55], [56].

## Supporting Information

Figure S1 (A) NMR signal crowding in the amide-aliphatic region of a [^1^H-^1^H]-NOESY spectrum of the cytosolic domain of Hrk (Hrk-ΔTM) in the presence of 35% (v/v) TFE. (B, C) Amide region of 1D ^1^H-NMR spectra of Hrk-ΔTM in the absence (B) and presence (C) of 35% (v/v) TFE.
(PDF)

Figure S2 (A-D) Amide and aliphatic regions of 1D ^1^H-NMR spectra of Hrk-22_53 without TFE (A, B) and with TFE (C, D). (E) Amide region of a [^1^H-^1^H]-NOESY spectrum of Hrk-22_53 in water without TFE showing H^N^-H^N^ (i, i+1) NOEs typical of the helical conformation.
(PDF)

Figure S3 Difference between observed ^1^H_α_ chemical shifts and tabulated random coil values from reference [27] vs. residue number for Hrk-22_53 in the presence (black) and absence (white) of TFE. For Gly 24 and Gly 41 the difference relative to the random coil value is represented for the largest ^1^H_α_ chemical shift. The large deviations of the first residue result from N-terminal effects caused by the positive charge of the amide group.
(PDF)

Figure S4 (A, B) Amide-aliphatic region of [^1^H-^1^H]-NOESY spectra of Hrk-TM in micelles (A) and the protein FSD [58] in water (B). The thick line between 4.4 and 4.6ppm represents the water chemical shift, which is different in (A) and (B) because NOESY spectra were acquired at different temperature. (C) Amide and aliphatic regions of 1D ^1^H-NMR spectra of Hrk-TM in micelles.
(PDF)

Table S1 Structural statistics of Hrk-22_53 in TFE.
(DOC)

Table S2 Structural statistics of Hrk-TM in micelles.
(DOC)

## References

[1] Kerr JF, Wyllie AH, Currie AR (1972). Apoptosis: a basic biological phenomenon with wide-ranging implications in tissue kinetics.. Br J Cancer.

[2] Li J, Yuan J (2008). Caspases in apoptosis and beyond.. Oncogene.

[3] Adams JM, Cory S (1998). The Bcl-2 protein family: arbiters of cell survival.. Science.

[4] Danial NN, Korsmeyer SJ (2004). Cell Death.. Cell.

[5] Oltvai ZN, Korsmeyer SJ (1994). Checkpoints of dueling dimers foil death wishes.. Cell.

[6] Reed JC (2000). Mechanisms of Apoptosis.. Am J Pathol.

[7] David CS, Huang DCS, Strasser A (2000). BH3-only proteins: essential initiators of apoptotic cell death.. Cell.

[8] Lomonosova E, Chinnadurai G (2009). BH3-only proteins in apoptosis and beyond: an overview.. Oncogene.

[9] Chou JJ, Li H, Salvesen GS, Yuan J, Wagner G (1999). Solution structure of BID, an intracellular amplifier of apoptotic signaling.. Cell.

[10] McDonnell JM, Fushman D, Milliman CL, Korsmeyer SJ, Cowburn D (1999). Solution structure of the proapoptotic molecule BID: a structural basis for apoptotic agonists and antagonists.. Cell.

[11] Hinds MG, Smits C, Fredericks-Short R, Risk JM, Bailey M (2007). Bim, Bad and Bmf: intrinsically unstructured BH3-only proteins that undergo a localized conformational change upon binding to prosurvival Bcl-2 targets.. Cell Death Differ.

[12] Bocharov EV, Pustovalova YE, Pavlov KV, Volynsky PE, Goncharuk MV (2007). Unique dimeric structure of BNip3 transmembrane domain suggests membrane permeabilization as a cell death trigger.. J Biol Chem.

[13] Sattler M, Liang H, Nettesheim DG, Meadows RP, Harlan JE (1997). Structure of Bcl-X_L_/Bak peptide complex: recognition between regulators of apoptosis.. Science.

[14] Petros AM, Nettesheim DG, Wang Y, Olejniczak ET, Meadows RP (2000). Rationale for Bcl-X_L_/Bad peptide complex formation from structure, mutagenesis and biophysical studies.. Protein Sci.

[15] Liu Q, Moldoveanu T, Sprules T, Matta-Camacho E, Mansur-Azzam N (2010). Apoptotic regulation by MCL-1 through hetero-dimerization.. J Biol Chem.

[16] Horne WS, Boersma MD, Windsor MA, Gellman SH (2008). Sequence-based design of α/β-Peptide foldamers that mimic BH3 domains.. Angew Chem Int Ed.

[17] Vogler M, Dinsdale D, Dyer MJ, Cohen GM (2009). Bcl-2 inhibitors: small molecules with a big impact on cancer therapy.. Cell Death Differ.

[18] Letai A, Bassik MC, Walensky LD, Sorcinelli MD, Weiler S (2004). Distinct BH3 domains either sensitize or activate mitochondrial apoptosis, serving as prototype cancer therapeutics.. Cancer Cell.

[19] Inohara N, Ding L, Chen S, Núñez G (1997). Harakiri, a novel regulator of cell death, encodes a protein that activates apoptosis and interacts selectively with survival-promoting proteins Bcl-2 and Bcl-X(L).. EMBO J.

[20] Bernabeu A, Guillén J, Pérez-Berná AJ, Moreno MR, Villalaín J (2007). Structure of the C-terminal domain of the proapoptotic protein Hrk and its interaction with model membranes.. Biochem Biophys Acta.

[21] Chen L, Willis SN, Wei A, Smith BJ, Fletcher JI (2005). Differential targeting of prosurvival Bcl-2 Proteins by their BH3-only ligands allows complementary apoptotic function.. Mol Cell.

[22] Sborgi L, Barrera-Vilarmau S, Obregón P, de Alba E (2010). Characterization of a novel interaction between Bcl-2 members Diva and Harakiri.. PLoS ONE.

[23] Rost B, Yachdav G, Liu J (2004). The PredictProtein Server.. Nucl Acids Res.

[24] Certo M, Del Gaizo Moore V, Nishino M, Wei G, Korsmeyer S (2006). Mitochondria primed by death signals determine cellular addiction to antiapoptotic Bcl-2 family members.. Cancer Cell.

[25] de Alba E, Jiménez MA, Rico M, Nieto JL (1996). Conformational investigation of designed short linear peptides able to fold into beta-hairpin structures in aqueous solution.. Fold Des.

[26] Bruix M, Muñoz V, Campos-Olivas R, del Bosque JR, Serrano L (1997). Characterisation of the isolated Che Y C-terminal fragment (79-129). Exploring the structure/stability/folding relationships of the α/β parallel protein Che Y.. Eur J Biochem.

[27] Wishart DS, Bigam CG, Holm A, Hodges RS, Sykes BD (1995). ^1^H, ^13^C and ^15^N random coil NMR chemical shifts of the common amino acids. I. Investigations of nearest-neighbor effects.. J Biomol NMR.

[28] Wüthrich K (1986). NMR of Proteins and Nucleic Acids..

[29] Lee EF, Sadowsky JD, Smith BJ, Czabotar PE, Peterson-Kaufman KJ (2009). High-resolution structural characterization of a helical alpha/beta-peptide foldamer bound to the anti-apoptotic protein Bcl-x_L_.. Angew Chemie Intl Ed.

[30] Uversky VN (2010). Seven lessons from one IDP structural analysis.. Structure.

[31] Shoemaker BA, Portman JJ, Wolynes PG (2000). Speeding molecular recognition by using the folding funnel: The fly-casting mechanism.. Proc Natl Acad Sci USA.

[32] Sugase K, Dyson HJ, Wright, PE (2007). Mechanism of coupled folding and binding of an intrinsically disordered protein.. Nature.

[33] Tompa P, Fuxreiter M (2008). Fuzzy complexes: polymorphism and structural disorder in protein–protein interactions.. Trends Biochem Sci.

[34] Ragusa MJ, Dancheck B, Critton DA, Nairn AC, Page R (2010). Spinophilin directs protein phosphatase 1 specificity by blocking substrate binding sites.. Nat Struct Mol Biol.

[35] Masotti L, Spinsni A, Urry DW (1980). Conformational studies on the gramicidin A transmembrane channel in lipid micelles and liposomes.. Cell Biophys.

[36] Pervushin KV, Orekhov VY, Popov AI, Musina LY, Arseniev AS (1994). Three-dimensional structure of (1-7l) bacterioopsin solubilized in methanol-chloroform and SDS micelles determined by ^l5^N-^l^H heteronuclear NMR spectroscopy.. Eur J Biochem.

[37] Schnell JR, Chou JJ (2008). Structure and mechanism of the M2 proton channel of influenza A virus.. Nature.

[38] Rath A, Johnson RM, Deber CM (2007). Peptides as transmembrane segments: decrypting the determinants for helix-helix interactions in membrane proteins.. Biopolymers.

[39] Spera S, Bax A (1991). Empirical correlation between protein backbone conformation and Cα and Cβ. ^13^C nuclear magnetic resonance chemical shifts.. J Am Chem Soc.

[40] Yau W-M, Wimley WC, Gawrish K, White SH (1998). The preference of tryptophan for membrane interfaces.. Biochemistry.

[41] Pinheiro AS, Marsh JA, Forman-Kay JD, Peti W (2011). Structural signature of the MYPT1-PP1 interaction.. J Am Chem Soc.

[42] de Alba E (2009). Structure and interdomain dynamics of apoptosis-associated speck-like protein containing a CARD (ASC).. J Biol Chem.

[43] Bogner C, Leber B, Andrews DW (2010). Apoptosis: embedded in membranes.. Curr Opin Cell Biol.

[44] Bax A, Grzesiek S (1993). Methodological advances in protein NMR.. Accounts Chem Res.

[45] Cavanagh J, Fairbrother WJ, Palmer AG, Rance M, Skelton NJ (2006).

[46] Bax A, Davis AG (1985). MLEV-17 based two-dimensional homonuclear magnetization transfer spectroscopy.. J Magn Reson.

[47] Delaglio F, Grzesiek S, Vuister GW, Zhu G, Pfeifer J (1995). NMRPipe: a multidimensional spectral processing system based on UNIX pipes.. J Biomol NMR.

[48] Garrett DS, Powers R, Gronenborn AM, Clore GM (1991). A common sense approach to peak picking in two-, three-, and four-dimensional spectra using computer analysis of contour diagrams.. J Magn Reson.

[49] Goddard TD, Kneller DG

[50] Nilges M (1993). A calculation strategy for the structure determination of symmetric dimers by ^1^H NMR.. Proteins.

[51] Cornilescu G, Delaglio F, Bax A (1999). Protein backbone angle restraints from searching a database for chemical shift and sequence homology.. J Biomol NMR.

[52] Schwieters CD, Kuszewski JJ, Tjandra N, Clore GM (2003). The Xplor-NIH NMR molecular structure determination package.. J Magn Reson.

[53] Koradi R, Billeter M, Wüthrich K (1996). MOLMOL: a program for display and analysis of macromolecular structures.. J Mol Graphics.

[54] Laskowski RA, Rullmann JA, MacArthur MW, Kaptein R, Thornton JM (1996). AQUA and PROCHECK-NMR: Programs for checking the quality of protein structures solved by NMR.. J Biomol NMR.

[55] Rohl CA, Chakrabartty A, Balwin RL (1996). Helix propagation and N-cap propensities of the amino acids measured in alanine-based peptides in 40 volume percent trifluoroethanol.. Protein Sci.

[56] Meyers JK, Pace CN, Scholtz JM (1997). Helix propensities are identical in proteins and peptides.. Biochemistry.

[57] Feller SE, Yin D, Pastor RW, MacKerell AD (1997). Molecular dynamics simulation of unsaturated lipids at low hydration: Parametrization and comparison with diffraction studies.. Biophys J.

[58] Sadqi M, de Alba E, Pérez-Jiménez R, Sánchez-Ruíz JM, Muñoz V (2009). A designed protein as experimental model of primordial folding.. Proc Natl Acad Sci USA.

