# Supplementary figures and images for: Intrinsic Order and Disorder in the Bcl-2 Member Harakiri: Insights into Its Proapoptotic Activity
Source: PLoS One. 2011 Jun 23;6(6):e21413. doi: 10.1371/journal.pone.0021413 (PMC3121775; doi:10.1371/journal.pone.0021413)

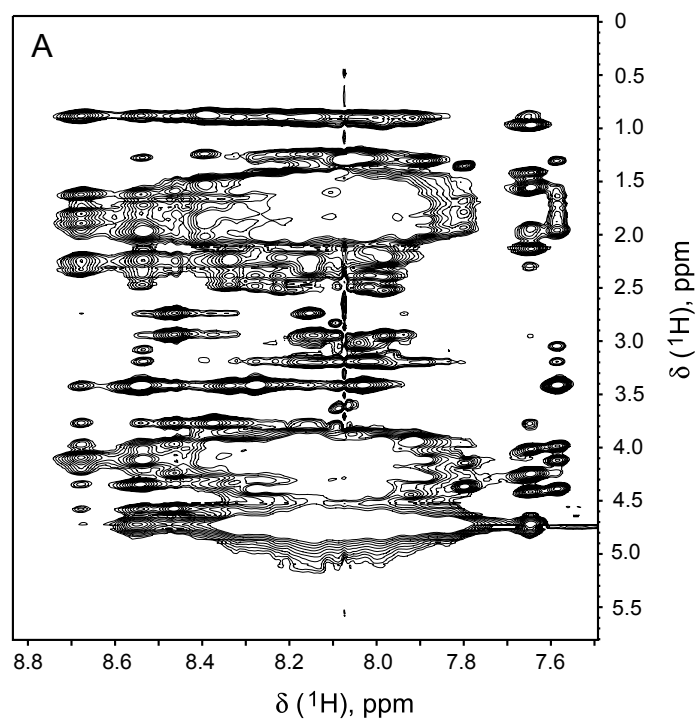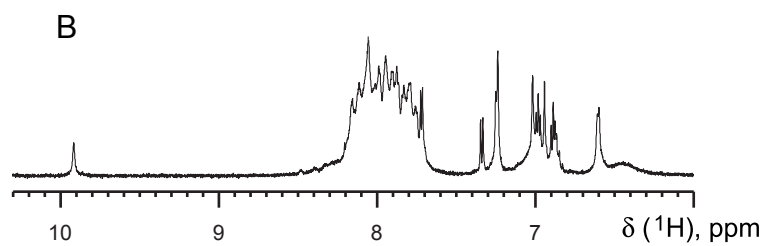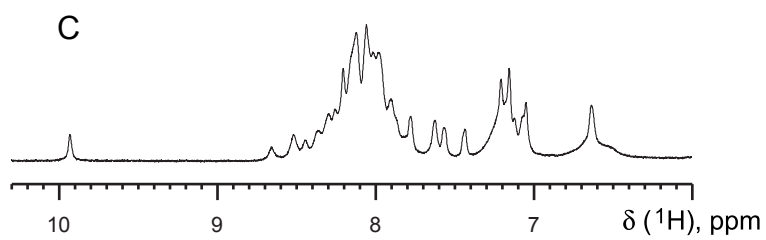

Supplement: Figure S1 — (A) NMR signal crowding in the amide-aliphatic region of a [1H-1H]-NOESY spectrum of the cytosolic domain of Hrk (Hrk-ΔTM) in the presence of 35% (v/v) TFE. (B, C) Amide region of 1D 1H-NMR spectra of Hrk-ΔTM in the absence (B) and presence (C) of 35% (v/v) TFE. (PDF) [file pone.0021413.s001.pdf]

A

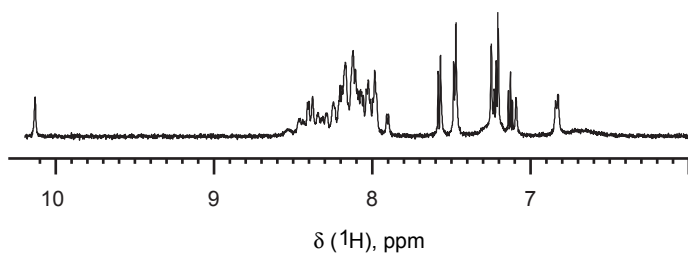

B

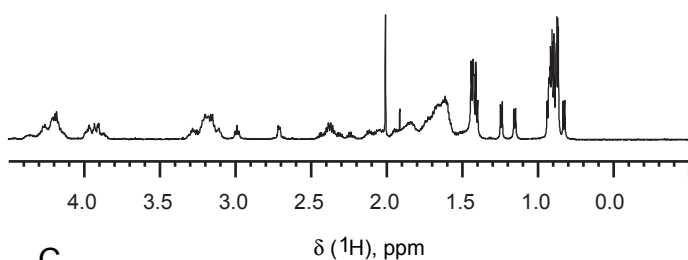

C

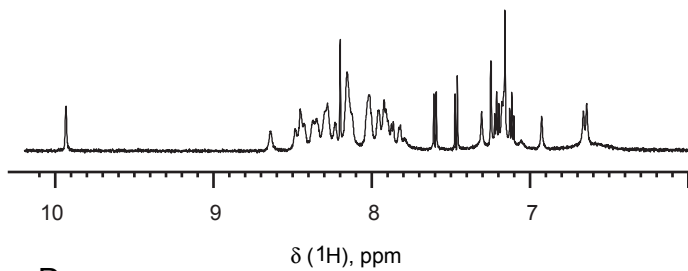

D

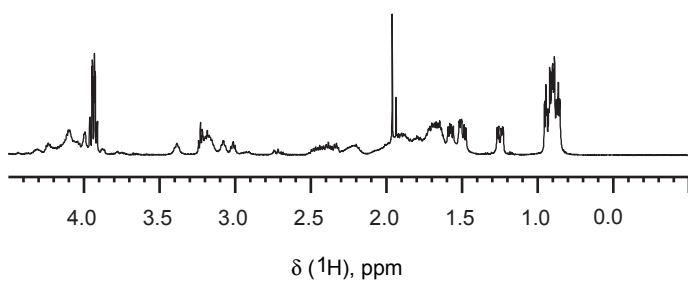

E

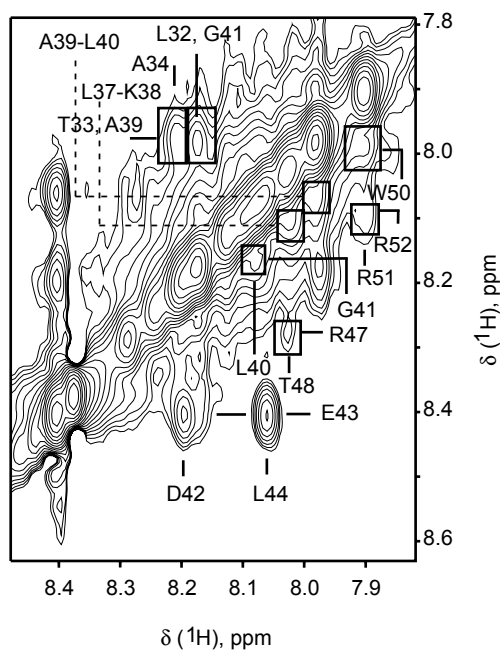

Supplement: Figure S2 — (A-D) Amide and aliphatic regions of 1D 1H-NMR spectra of Hrk-22_53 without TFE (A, B) and with TFE (C, D). (E) Amide region of a [1H-1H]-NOESY spectrum of Hrk-22_53 in water without TFE showing HN-HN (i, i+1) NOEs typical of the helical conformation. (PDF) [file pone.0021413.s002.pdf]

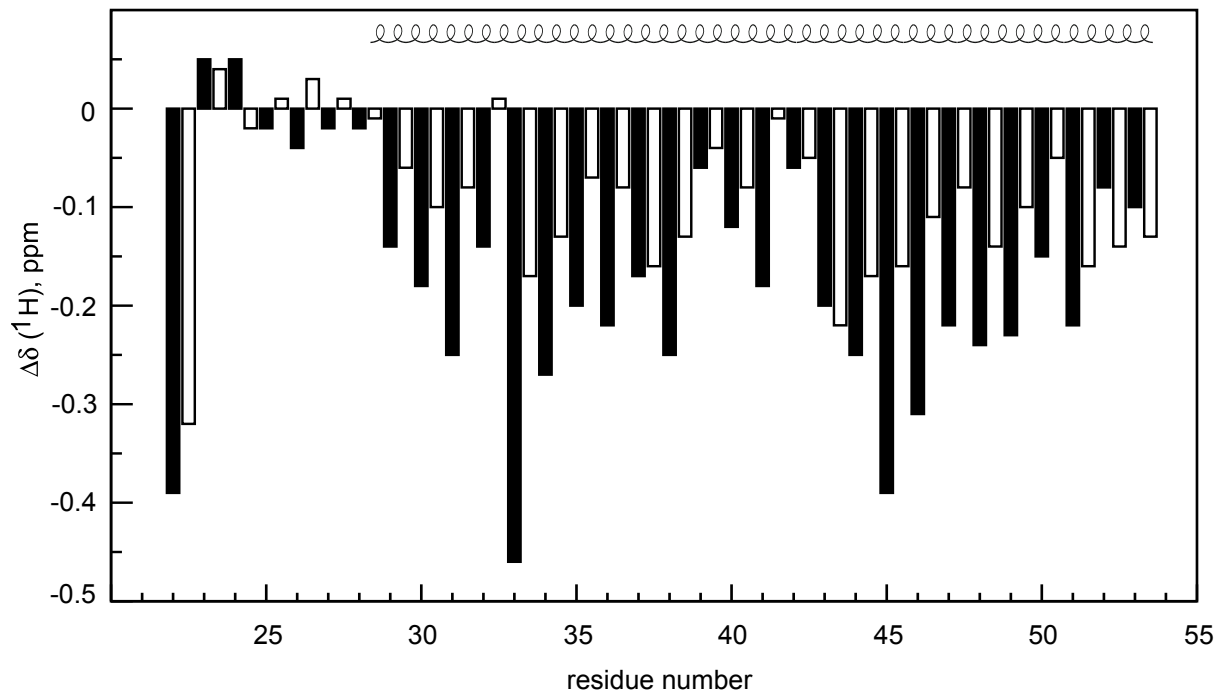

Supplement: Figure S3 — Difference between observed 1Hα chemical shifts and tabulated random coil values from reference [27] vs. residue number for Hrk-22_53 in the presence (black) and absence (white) of TFE. For Gly 24 and Gly 41 the difference relative to the random coil value is represented for the largest 1Hα chemical shift. The large deviations of the first residue result from N-terminal effects caused by the positive charge of the amide group. (PDF) [file pone.0021413.s003.pdf]

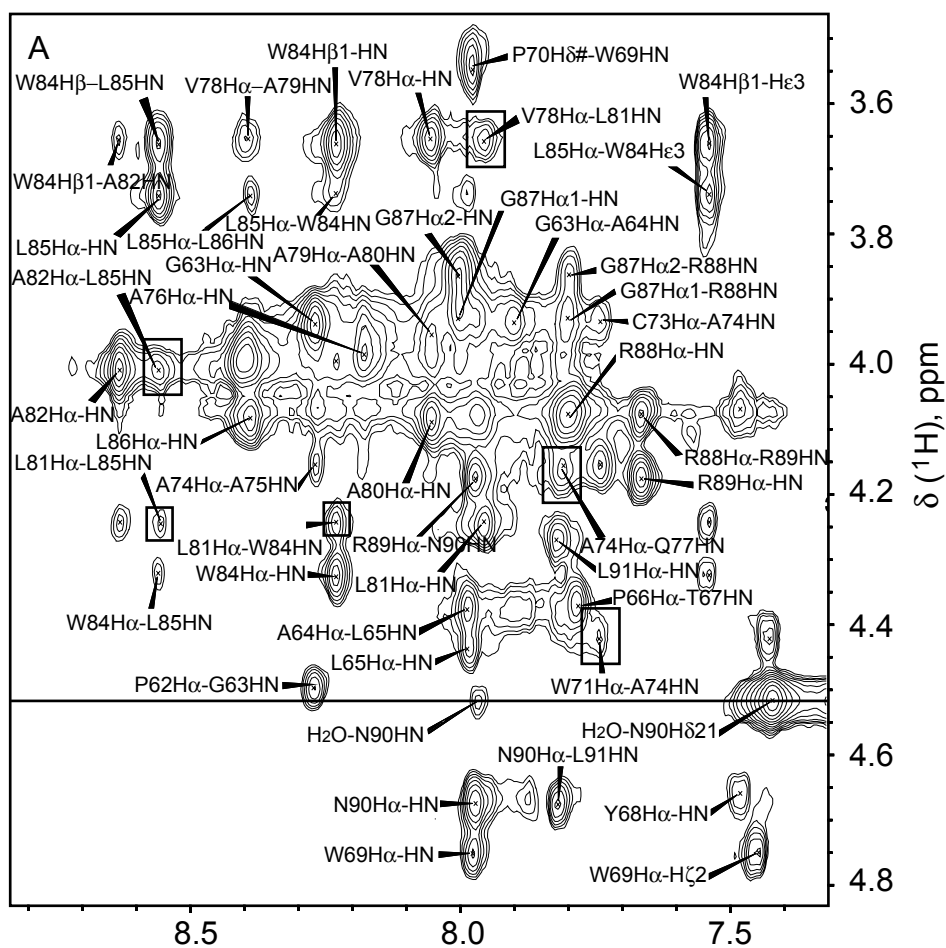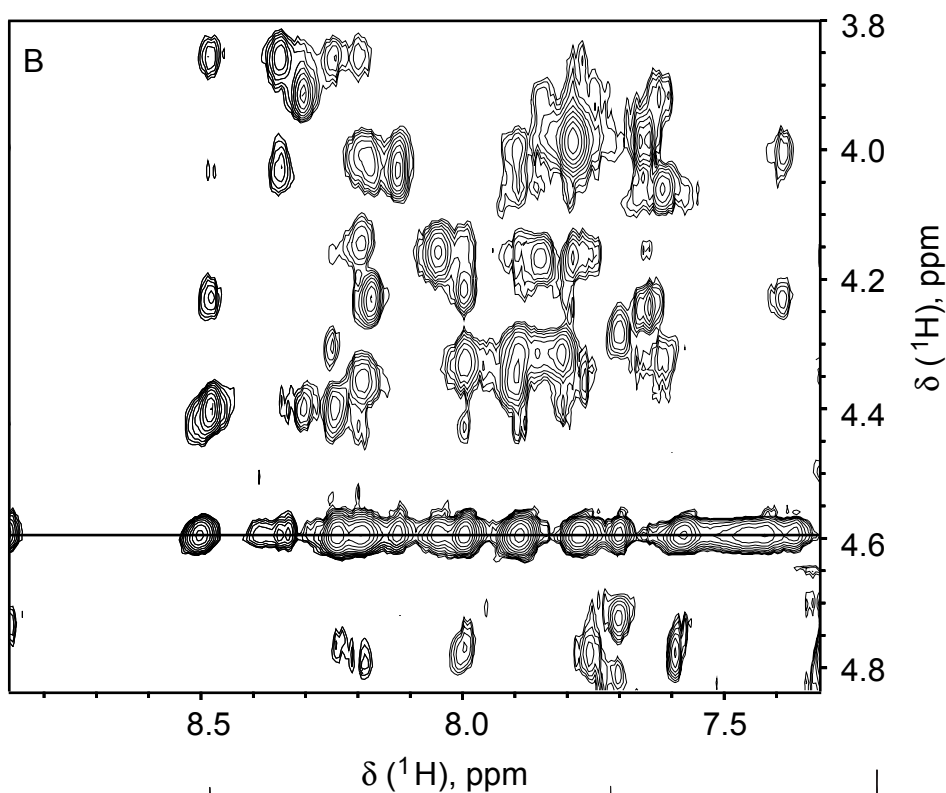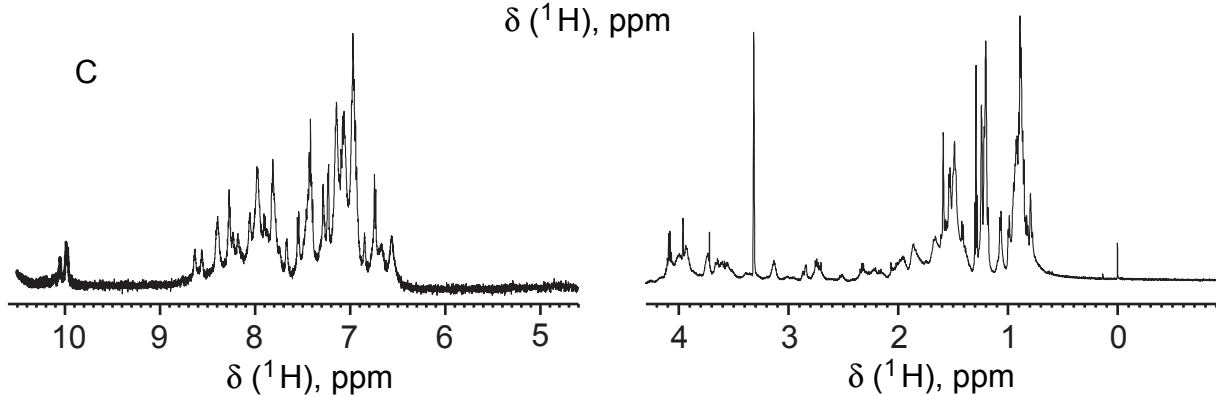

Supplement: Figure S4 — (A, B) Amide-aliphatic region of [1H-1H]-NOESY spectra of Hrk-TM in micelles (A) and the protein FSD [58] in water (B). The thick line between 4.4 and 4.6ppm represents the water chemical shift, which is different in (A) and (B) because NOESY spectra were acquired at different temperature. (C) Amide and aliphatic regions of 1D 1H-NMR spectra of Hrk-TM in micelles. (PDF) [file pone.0021413.s004.pdf]
