# Supplementary material for: Intrinsic Order and Disorder in the Bcl-2 Member Harakiri: Insights into Its Proapoptotic Activity
Source: PLoS One. 2011 Jun 23;6(6):e21413. doi: 10.1371/journal.pone.0021413 (PMC3121775; doi:10.1371/journal.pone.0021413)
Supplement: Table S1 — Structural statistics of Hrk-22_53 in TFE. (DOC) [file pone.0021413.s005.doc]

Table S1. Structural statistics of Hrk-22_53 in TFE*

| **Restraints** | **r.m.s.deviations** | |
| --- | --- | --- |
|  | **20 lowest-energy conformers** | **Lowest energy conformer** |
| **Distances, Å (309)** | | |
| Intra-residue (159) | | |
| Sequential |i–j|=1 (73) | | |
| Short-range |i–j|≤5 (77) | 0.017 ± 0.002 | 0.019 |
| Long-range |i–j|≥5 (0) | | |
| **Hydrogen bonds, Å (17)** | 0.008 ± 0.003 | 0.002 |
| **Dihedrals (φ ψ, º) (38)** | 0.31 ± 0.04 | 0.33 |
| **Deviations form ideal covalent geometry** | | |
| Bonds, Å | 0.0047 ± 0.0003 | 0.0041 |
| Angles, º | 0.43 ± 0.02 | 0.39 |
| Impropers, º | 0.3 ± 0.1 | 0.2 |
| **Structure quality** | | |
| Lennard-Jones potential energy (Kcal mol^-1^) † | -131 ± 3 | -134 |
| Ramachandran # | 96.7% (residues in most favored regions) | |
| (residues 28-51) | 0.7% (residues in disallowed regions) | |
| **Coordinate precision, Å** | Residues 28-51 | |
| Backbone heavy atoms | 0.5 ± 0.1 | |
| All heavy atoms | 1.2 ± 0.2 | |

*Statistics were calculated for the 20 conformers with the lowest overall energies and no NOE or dihedral angle restraint violations greater than 0.3Å and 3.0˚, respectively.

† The Lennard-Jones van der Waals energy was calculated with the CHARMM PARAM19/20 parameters and was not included in structure calculation.

# Calculated with PROCHECK-NMR [54].
