# Supplementary material for: Intrinsic Order and Disorder in the Bcl-2 Member Harakiri: Insights into Its Proapoptotic Activity
Source: PLoS One. 2011 Jun 23;6(6):e21413. doi: 10.1371/journal.pone.0021413 (PMC3121775; doi:10.1371/journal.pone.0021413)
Supplement: Table S2 — Structural statistics of Hrk-TM in micelles. (DOC) [file pone.0021413.s006.doc]

Table S2. Structural statistics of Hrk-TM in micelles*

| **Restraints** | **r.m.s.deviations** | |
| --- | --- | --- |
|  | **20 lowest-energy conformers** | **Lowest energy conformer** |
| **Distances, Å (444)** | | |
| Intra-residue (214) | | |
| Sequential |i–j|=1 (114) | | |
| Short-range |i–j|≤5 (116) | 0.079 ± 0.002 | 0.080 |
| Long-range |i–j|≥5 (0) | | |
| **Hydrogen bonds, Å (12)** | 0.077 ± 0.001 | 0.078 |
| **Dihedrals (φ ψ, º) (32)** | 0.35 ± 0.05 | 0.32 |
| **Deviations form ideal covalent geometry** | | |
| Bonds, Å | 0.0068 ± 0.0001 | 0.0067 |
| Angles, º | 0.84 ± 0.01 | 0.81 |
| Impropers, º | 0.33 ± 0.01 | 0.31 |
| **Structure quality** | | |
| Lennard-Jones potential energy (Kcal mol^-1^) † | -107 ± 3 | -104 |
| Ramachandran # | 86.5% (residues in most favored regions) | |
| (residues 28-51) | 0% (residues in disallowed regions) | |
| **Coordinate precision, Å** | Residues 69-91 | |
| Backbone heavy atoms | 0.3 ± 0.1 | |
| All heavy atoms | 0.6 ± 0.1 | |

*Statistics were calculated for the 20 conformers with the lowest overall energies and no NOE or dihedral angle restraint violations greater than 0.5Å and 5.0˚, respectively.

† The Lennard-Jones van der Waals energy was calculated with the CHARMM PARAM19/20 parameters and was not included in structure calculation.

# Calculated with PROCHECK-NMR [54].
